# Effects of Mantra Meditation versus Music Listening on Knee Pain, Function, and Related Outcomes in Older Adults with Knee Osteoarthritis: An Exploratory Randomized Clinical Trial (RCT)

**DOI:** 10.1155/2018/7683897

**Published:** 2018-08-30

**Authors:** Kim E. Innes, Terry Kit Selfe, Sahiti Kandati, Sijin Wen, Zenzi Huysmans

**Affiliations:** ^1^Department of Epidemiology, School of Public Health, West Virginia University, Morgantown, WV, USA; ^2^Department of Biomedical and Health Information Services, Health Science Center Libraries, University of Florida, Gainesville, FL, USA; ^3^School of Dentistry, SUNY-Buffalo, Buffalo, NY, USA; ^4^Department of Biostatistics, School of Public Health, West Virginia University, Morgantown, WV, USA; ^5^College of Physical Activity and Sport Sciences, West Virginia University, Morgantown, WV, USA

## Abstract

**Objective:**

Disease-modifying treatments for OA remain elusive, and commonly used medications can have serious side effects. Although meditation and music listening (ML) have been shown to improve outcomes in certain chronic pain populations, research in OA is sparse. In this pilot RCT, we explore the effects of two mind-body practices, mantra meditation (MM) and ML, on knee pain, function, and related outcomes in adults with knee OA.

**Methods:**

Twenty-two older ambulatory adults diagnosed with knee OA were randomized to a MM (N=11) or ML program (N=11) and asked to practice 15-20 minutes, twice daily for 8 weeks. Core outcomes included knee pain (Knee Injury and Osteoarthritis Outcome Score [KOOS] and Numeric Rating Scale), knee function (KOOS), and perceived OA severity (Patient Global Assessment). Additional outcomes included perceived stress (Perceived Stress Scale), mood (Profile of Mood States), sleep (Pittsburgh Sleep Quality Index), and health-related quality of life (QOL, SF-36). Participants were assessed at baseline and following completion of the program.

**Results:**

Twenty participants (91%) completed the study (9 MM, 11 ML). Compliance was excellent; participants completed an average of 12.1±0.83 sessions/week. Relative to baseline, participants in both groups demonstrated improvement post-intervention in all core outcomes, including knee pain, function, and perceived OA severity, as well as improvement in mood, perceived stress, and QOL (Physical Health) (p's≤0.05). Relative to ML, the MM group showed greater improvements in overall mood and sleep (p's≤0.04), QOL-Mental Health (p<0.07), kinesiophobia (p=0.09), and two domains of the KOOS (p's<0.09).

**Conclusions:**

Findings of this exploratory RCT suggest that a simple MM and, possibly, ML program may be effective in reducing knee pain and dysfunction, decreasing stress, and improving mood, sleep, and QOL in adults with knee OA.

## 1. Introduction

Osteoarthritis (OA) is the most common form of arthritis and a leading cause of chronic pain and disability in the U.S., affecting at least 30.8 million American adults [1]. OA is also associated with increased risk for medical comorbidity [2, 3], falls [4], and mortality [2, 5, 6] and with significant deterioration in mood [7, 8], sleep quality [7, 9], and quality of life (QOL) [5]. OA of the knee, the joint most commonly affected in OA patients, accounts for 83% of total OA burden [10, 11]. Approximately 45% of adults are projected to develop symptomatic knee OA by age 85, and an estimated two-thirds of those who are obese will develop symptomatic knee OA in their lifetime [12].

There is currently no cure for OA, and there are no effective treatments available for modifying the course of the disease [13]. Rather, current recommended treatment approaches focus on symptom management and functional restoration [13]. Although pharmaceuticals remain the treatment mainstay for the vast majority of patients with OA [14, 15], medications used to alleviate OA pain can be costly and carry substantial side effects that are both more common and more problematic in older adults [16–19]. For example, while the existing literature and guideline recommendations do not support the use of opioids for the management of knee OA, these medications are frequently used [15, 20]. A recent analysis of U.S. commercial and Medicare claims data from over 6 million privately insured patients aged 40-75 years (2009-15) indicated that opioid use is elevated among those with knee OA (33-35%) and that likelihood of narcotic use was 6- to 8-fold higher in this population relative to those without diagnosed knee OA [20].

Of particular concern is the trend of increasing opioid prescription for OA during the past decade. For example, opioid use among U.S. adults 65 and older with knee OA rose from 31% in 2003 to 40% in 2009 [13], despite overwhelming evidence that opioid therapy for chronic noncancer pain in older adults carries significant risks. Notably, these medications are addictive, carry serious side effects, can increase risk for falls, disability, and all-cause mortality, and are ineffective for long-term pain control in most [ca 75%] OA patients [19, 21–23].

The American College of Rheumatology clinical guidelines recommend the use of nonpharmacologic therapies for first-line management of knee OA [24]. Moreover, the Osteoarthritis Research Society International (OARSI) practice guidelines emphasize patient-driven therapies and self-help strategies in the initial stages of OA management [25]. Identifying safe, sustainable self-management interventions that are effective in addressing not only pain and dysfunction, but the associated impairment in mood, sleep, and quality of life is of clear importance.

Movement-based mind-body therapies such as yoga and tai chi, as well as conventional physical exercise, have been shown to decrease pain and improve physical function in older adults with OA [22]. There is also growing evidence that meditation and music-based interventions, including simple, passive music listening, may reduce pain [26–29], improve mood [26–30], and enhance QOL [28, 29] in adults with OA and other musculoskeletal [26–28] and chronic noncancer pain conditions [27, 28, 30]. In our recent uncontrolled trial of mantra meditation in older adults with knee OA, participants showed marked and significant improvements in pain and physical function, mood, and a proxy measure for sleep following completion of a simple 8-week mantra meditation program [31]. However, despite the promise and apparent therapeutic potential of these simple therapies, rigorous studies regarding the effects of meditation or music in adults with OA are lacking. Building on our promising preliminary findings, this exploratory randomized clinical trial (RCT) compared the effects of a mantra meditation versus a music listening program on pain, function, and related psychosocial factors in older adults with symptomatic OA of the knee.

## 2. Subjects and Methods

In this community-based pilot RCT, we investigated the effects of a simple mantra meditation (MM) program versus a music listening (ML) program on knee pain, function, and related indices in 22 ambulatory older adults with physician-confirmed OA of the knee.

### 2.1. Study Participants: Participant Recruitment, Characteristics, Screening, and Enrollment

The study was approved by the West Virginia University Institutional Review Board. Independently living, ambulatory adults aged ≥50 years with OA were recruited using flyers and brochures posted in community, healthcare, and workplace settings, and advertisements posted on the university intranet and listservs. Study eligibility criteria included: at least 50 years of age; physician-confirmed diagnosis of OA of the knee; knee pain for at least 6 months, rated as moderately severe or worse (defined as a score > 3 on an 11-point numeric pain rating scale) for most days in the month prior to enrollment; and willingness and ability to abide by the protocol. Exclusion criteria were as follows: began or stopped medications, physical therapy, or supplements for the knee within 2 months preceding study enrollment; intra-articular corticosteroid or hyaluronic acid injection into the knee within 3 months preceding the study; significant injury to the knee within the past 6 months; arthroscopy of the knee within the past year; use of assistive devices other than a cane or knee brace; presence of an uncontrolled comorbid condition affecting the knee (e.g., rheumatoid arthritis); disease of the spine or other lower-extremity joints or poor general health interfering with compliance or assessment; a regular meditation practice within the past year; and/or history of psychotic or schizophrenic episodes.

Potential participants provided written informed consent and underwent a full screening and baseline assessment at the WVU Health Research Center. We enrolled participants on a rolling basis in two waves over a total of 6 months in 2015-16. Upon completion of the 8-week program, participants returned for follow-up assessments (see below). All participants were encouraged to continue stable use of any supplements/medications that were currently being taken for knee pain and to avoid beginning any new drugs or treatments for knee pain during the study period.

### 2.2. Randomization

Following provision of consent and confirmation of eligibility and collection of baseline data (see below), participants were randomized to the meditation or music listening group, based on an allocation sequence generated by the study statistician and using a block randomization method to ensure equal distribution between treatment groups. The statistician, who had no contact with the participants, generated an assignment master list and provided sequentially numbered opaque envelopes containing the group assignment. The consenting team member gave the next envelope in sequence to the participant. The participant opened the envelope to discover his/her intervention group assignment.

### 2.3. Interventions

#### 2.3.1. Training

Following randomization, each participant received 30-45 minutes of in-person training in his/her respective program and received a brief, illustrated reference guide, a program CD, and a portable CD player for home use. Each program CD included 15-minute and 20-minute tracks. The training was provided by a team member familiar with both programs and experienced in teaching a variety of relaxation techniques; training included presentation of the instructions for each program (described below), introduction to the operation of the CD player and various CD tracks, and use of the practice log. The participant then performed their first practice session and recorded it on the log sheet while the trainer observed and provided any guidance required by the participant to perform the intervention at home with proficiency. In addition, the trainer followed up with each participant by phone during the first week of the study, and periodically thereafter as needed to address any concerns or questions arising during the course of the trial.

Both interventions entailed sitting comfortably, eyes closed, for 15-20 minutes twice daily every day for 8 weeks (112 sessions total) and documenting each session, including any comments, daily on the practice log provided. All participants were encouraged to begin the program with the 15-minute version and move on to the 20-minute version when they felt comfortable. All participants were instructed to select a quiet environment where there would be no disturbances for approximately 20 minutes (see below). Upon completing the 15-20-minute session, participants were instructed to take as much time as necessary to gently return to full alertness before standing up and resuming normal activities.

#### 2.3.2. Mantra Meditation Program (MM)

The meditation technique was a simple, easy-to-learn mantra meditation practice. A list of possible mantras (sounds or words) was provided in the instruction sheet. Each participant was instructed to select a mantra that appealed to him/her based on the sound or vibrational quality, and to avoid mantras that might precipitate trains of thought or emotional responses. The meditation CD contained both guided and silent sessions, including soft chimes to announce the beginning and end of a 15-20-minute session. Participants were instructed to take a few deep breaths, releasing any stress or tension during exhalation, then to begin silently repeating the chosen mantra, gently letting go of all other thoughts for 15–20 minutes. Emphasis was placed on the practice being easy and effortless. After 15–20 minutes had passed, the participants were to stop repeating their mantras and sit quietly for approximately 2 minutes before opening their eyes.

#### 2.3.3. Music Listening Program (ML)

The ML program CD contained selections of relaxing instrumental music from each of six composers, including Mozart, Bach, Vivaldi, Beethoven, Pachelbel, and Debussy (a total of 15 tracks). Participants were allowed to choose which musical selections to listen to on a daily basis but were asked to try each composer at least once during the study.

### 2.4. Measures and Assessment

All participant assessments were performed by research staff blinded to participant treatment assignment.

#### 2.4.1. Baseline Data

These data were collected following provision of written informed consent. Information gathered included that on demographics; lifestyle factors (alcohol consumption, smoking status, caffeine consumption, and engagement in physical activity); body mass index (BMI, calculated as height(m)/weight(kg)^2^); and medical history, including current use of medications and supplements. At* follow-up*, participants were also specifically queried regarding any changes during the study period in medication and/or supplement use; caffeine or alcohol consumption, smoking status, or physical activity.

#### 2.4.2. Outcomes

All outcomes were assessed at baseline and within 2 weeks following completion of the 8-week intervention.* Core outcomes* included knee pain, assessed using the Knee Injury and Osteoarthritis Outcome Score [KOOS] [32] and the Numeric Rating Scale [NRS] [33], knee function (KOOS), and perceived OA severity (Patient Global Assessment) [34] as consistent with the OMERACT recommended core set outcomes [35]. All scales are reliable, well-validated instruments widely used for evaluating knee OA and shown to be sensitive to change with behavioral and other nonpharmacologic interventions [36–40].

The KOOS is a self-administered, condition-specific questionnaire developed as an extension of the Western Ontario and McMaster Universities Osteoarthritis Index (WOMAC). In addition to the 17-item subscale to assess knee function in activities of daily living (ADL) (equivalent to the WOMAC knee function subscale), the KOOS includes an expanded 9-item pain subscale and 7-item "other symptoms" subscale that incorporate, respectively, the WOMAC knee pain (5 items) and knee stiffness (2 items) subscales; the KOOS also includes two additional subscales to evaluate function in recreational/sport activities and knee-related quality of life (QOL). Higher scores indicate worse outcomes. Extensive psychometric testing has shown the KOOS to be a valid, reliable, and responsive instrument in a range of populations and across multiple languages [37, 41]. As aggregate scores are not recommended in scoring the KOOS [42], the total score (KOOS-WOMAC total) was calculated as the sum of the WOMAC pain, stiffness, and function subscale scores as per WOMAC scoring guidelines [43]. An 11-point NRS (ranging from 0 (“no pain”) to 10 (“worst pain possible”)) was used to rate current, average, least, and worst pain for the previous week. NRS scales have shown excellent reliability and validity for a range of populations, are easier to use, and have shown higher compliance and greater responsiveness to change with treatment than the visual analog scale (VAS), especially in older adults [44–48].

*Additional outcomes* included OA-related quality of life, symptoms, and function in leisure activities/sports, assessed using the KOOS subscales for these domains. In addition, we evaluated perceived stress (10-item Perceived Stress Scale (PSS) [49]), mood (65-item Profile of Mood States [50]), well-being (Psychological Well-Being Scale (PWBS) [51]), sleep quality (Pittsburgh Sleep Quality Index (PSQI) [52]), and health-related quality of life (36-item MOS Short Form-36 (SF-36) [51]). These self-report measures are established, well-validated instruments that have been used in a broad range of populations, including those with OA [53–56]. We also measured pain-related catastrophizing using the 13-item Pain Catastrophizing Scale (PCS) [57] and fear of movement using the 6-item Tampa Scale for Kinesiophobia (TSK-6) [58] to assess change over time in these potential mediators.

#### 2.4.3. Treatment Expectancy, Adherence, and Participant Satisfaction

To assess* expectation of benefit,* participants completed an abridged Credibility/Expectancy Questionnaire (CEQ) following their first intervention practice session; derived from the original 6-item questionnaire [59], the CEQ included 2 items scored on a 0-10 scale: "How confident are you this program will be beneficial?" and "What is the degree of improvement that you expect from this relaxation program?". Participants were provided with home practice logs to complete daily, recording the time and any comments regarding the daily session; practice logs were collected at the follow-up assessment. Finally, upon completion of the 8-week intervention or leaving the study, participants completed an exit questionnaire adapted from that used in our previous trials [31, 60–62] and including both structured and open-ended questions regarding the participants' experience with the study, perceived benefits and problems with the interventions, barriers to adherence, and other concerns.

### 2.5. Data Analysis

All data analyses were performed using IBM SPSS for Windows, Version 23. Differences in baseline characteristics by intervention group assignment were assessed using chi square (for categorical variables), Student's independent samples t-tests (for continuous variables with a normal distribution), or Mann–Whitney U tests (for ordinal or continuous variables with evidence of skewing). Potential differences between treatment groups in treatment expectancies, retention, and adherence were analyzed using chi square (attrition) and one-way ANOVA (adherence, treatment expectancies). In preliminary assessments, within-group changes over time at 8 weeks were assessed using ANCOVA with baseline scores as covariates; between-group differences in treatment outcomes were assessed using Repeated Measures ANOVA, with factors that differed at baseline (p<0.1) included as covariates. Variables with a nonnormal distribution were log-transformed for analysis, using the addition of a constant in the case of zero or negative values. We used multiple imputation to replace any missing data in our intention-to-treat (ITT) analyses [63, 64]. Effect sizes were calculated using Cohen's* d*. As this was an exploratory study designed to assess feasibility and to evaluate preliminary efficacy for a range of interrelated outcomes of direct clinical relevance to OA management, the alpha was set at 0.05 (two-sided) and we did not adjust for multiple comparisons. While a cut-point of 0.05 can be considered conservative given the small sample size and objectives of this exploratory study [65–67], we provide exact p values, along with effect sizes, point estimates, and measures of variability to allow readers to draw their own conclusions regarding the findings.

To assess the potential relationship of treatment expectancy scores and practice adherence to change over time in knee pain, function, and related outcomes, as well as to changes in mood, sleep, well-being, stress, QOL, fear of movement, and pain catastrophizing, bivariate and age- and sex-adjusted correlations were performed using Pearson product-moment correlation. To evaluate the potential influence of treatment expectancy on change over time in core outcomes, we also conducted additional analyses adjusting for this factor.

## 3. Results

Twenty-two eligible adults with symptomatic OA of the knee were enrolled in the study. As illustrated in Table 1, study participants were predominantly non-Hispanic white (82%) and female (68%), with an average age of 58.5±1.4 (range 50-74) years. Engagement in physical activity was low overall, with over 40% reporting none at all, and 68% indicating less than the recommended 150 minutes/week. BMI averaged 34±1.5, and prevalence of comorbidity was high in this sample; 82% of participants reported at least one, and 45% indicated at least 2 chronic comorbid conditions (Table 1). Common comorbid conditions included obesity (68%) and hypertension (68%). Prescription medication use was also high, with 86% reporting using 1-2 and almost 60% at least 3 medications. Most (68%) were on analgesics, with 32% using opioids or muscle relaxants and 59% reporting regular use of NSAIDs. Clinically significant sleep impairment, defined as PSQI>5 [52, 68], was present in over 95% of participants at baseline, and psychosocial measures indicated high baseline levels of distress in this population (Table 2). Treatment expectancy scores indicated positive expectations overall, with both items averaging over 7 on a scale of 1-10 (means ± SE=7.8±0.3, 7.2±0.4).

Participants in the MM group averaged higher baseline BMI and were more likely to indicate absence of physical activity and to report higher analgesic (and specifically NSAID) use than those of the ML group. The two groups did not differ significantly in other demographic characteristics, lifestyle factors, medical history, or medication use (Table 1). Likewise, there were no significant between-group differences in baseline scores on core or secondary outcome measures (Table 2) or in measures of kinesiophobia or pain catastrophizing (p's >0.1).

Each participant received the intervention as allocated. Participant retention was high, with 20/22 (91%) participants (9/11 MM, 11 ML) completing the 8-week intervention. Drop-out occurred early in the study, with one participant withdrawing in week one due to a job change and the second in week two due to an injury unrelated to the intervention. Adherence was also high, with participants completing an average of 94% of the 112 possible sessions (91% MM, 96% ML) and an average of 13.1±0.4 sessions/week (12.8±8 MM, 13.5±0.3 ML). There were no significant between-group differences in adherence (p's≥0.4). Similarly, there were no significant differences between the two groups in treatment expectancy (p's≥0.7). Treatment expectancy scores were* negatively* related to improvements over time in one measure of pain (NRS, average pain), overall mood, depression, and quality of life (r's ranging from -0.4 to -0.6, p's<0.05). No adverse events were observed or reported.

Responses on the exit questionnaires (N=20) also indicated overall high satisfaction with the study and study interventions. Eighty percent of participants (78% MM, 82% ML) indicated that they were likely or very likely to continue practicing. In response to the question “What did you like most about the study?", 65% (78% MM, 55% ML) reported they enjoyed taking time for themselves; 80% (78% MM, 82% ML) indicated that they found the practice to be soothing, calming, and/or relaxing; a number also noted their practice to help with pain (40%) and/or sleep (30%). Several (4 MM, 1 ML) noted experiencing increased focus, clarity, and/or awareness. In response to questions regarding challenges/barriers experienced in the study or program, 7 participants (3 MM, 4 ML) indicated no difficulties/barriers; others noted difficulty finding time to complete the practice (3 MM, 4 ML), with some (N=4) noting they felt the practice was too or a bit too long.

### 3.1. Change over Time in Knee Pain-Related Outcomes, Psychological Status, Sleep Quality, and Quality of Life

As illustrated in Table 3, participants in both groups demonstrated improvement at 8 weeks in primary outcomes, including knee pain (KOOS, p's≤0.03; NRS, p's<0.05) and perceived OA severity (PGA, p's≤0.04), as well as in current, average, and least knee pain (NRS, p's≤0.03). In addition, the MM group demonstrated significant gains in knee function (p<0.02), sport and recreation-related function (p=0.04), and knee-related quality of life (p<0.03). Although both effect sizes and absolute improvements in most measures were substantially larger in the MM than in the ML group, between-group differences were marginally significant for only the overall total (WOMAC) score and two KOOS subscales, sport/recreational function and knee-related QOL (Table 3). Using the OARSI/OMERACT criteria for treatment response in clinical trials of OA [69], the percentage of responders was also greater in the MM than in the ML group (67% versus 54%, respectively), but differences were not statistically significant.

Participants in both the MM and ML group also demonstrated improvements in perceived stress (p's ≤0.04) and QOL (Physical Health Component, p<0.01), as well as reductions in fear of movement (TKS, p≤0.05) and pain catastrophizing (PCS, MM, p<0.09, ML p<0.01) (Table 3). In addition, the MM group showed significant improvements in sleep quality (p<0.02 for overall, p's≤0.03 for sleep disturbance and daytime dysfunction), overall QOL-MH (p=0.01), overall mood (p<0.01), and multiple individual domains of both mood (confusion, depression, anger/hostility, and fatigue (p's≤0.04)) and QOL (energy/vitality, emotional well-being, and pain (p's≤0.04)).

Relative to ML, the MM group showed significantly greater improvements in overall sleep quality and mood (p's≤0.04) and in two individual mood domains, including tension/anxiety and anger/hostility (p's=0.01). Participants assigned to MM also tended to show greater gains in QOL-Mental Health (MH) (p<0.07), as well as in certain individual domains of mood (depression (p=0.06), confusion (p=0.09)) and QOL-PH (pain, general health (p's<0.09)).

ITT analyses using multiple imputation yielded similar results, as did analyses adjusting for baseline physical activity. Additional adjustment for treatment expectancy modestly strengthened the between-group differences in mood (overall and depression (p's≤0.03), sleep (p=0.02), and QOL-MH (p=0.04)) but did not otherwise appreciably alter findings in age-adjusted analyses. Three participants reported a change in medication, including two MM (lowered dose of ACE inhibitor (1), given a 3-day prescription for muscle relaxant for pain unrelated to OA (1)) and 1 ML participant (prescribed narcotic analgesic). Neither adjusting for change in medication nor eliminating these individuals from the analyses substantively altered the within-group or between-group findings.

### 3.2. Relation of Changes over Time in Knee Pain and Related Outcomes to Those in Psychological Status, Sleep Quality, Quality of Life, and Other Factors

As illustrated in part in Table 4, improvements in mood, both overall and in specific domains, were significantly correlated with declines in current pain (NRS, r's=0.5 overall; 0.4-0.5, tension, vigor; p's<0.05) and improvements in patients' assessment of their condition overall (PGA) (fatigue, r=0.65, p<0.005). Reductions in NRS measures of knee pain and OA-related symptoms (KOOS) were significantly correlated with several individual domains of sleep quality (r's=0.5-0.6, sleep latency, quality, duration, and disturbance) and with increases in mean hours of sleep (r's=0.5). Positive changes in mental and physical health-related quality of life, both overall and specific domains, were significantly correlated with improvements in several knee pain related outcomes, including overall knee-related symptoms and function (KOOS-WOMAC total score) and KOOS knee function (r's 0.5-0.6 overall; 0.4-0.5, individual domains (emotional well-being, energy/vitality, social function, pain, and physical function)), current and average knee pain (NRS, r's=0.4-0.5 overall; 0.5-0.7, emotional well-being, physical function), PGA (r's=0.4-5 overall and individual domains (emotional well-being, energy/vitality, social function physical function)), and knee-related QOL (r's=0.4-0.5 overall; 0.4-0.5, emotional well-being, physical function). In addition, decline in fear of movement was significantly correlated with improvements in average pain scores (r=0.6), OA severity (r=0.6), and all KOOS measures except knee pain (r's 0.4-0.6), supporting a possible mediating influence of kinesiophobia. Declines in pain catastrophizing (PCS) were correlated only with improvements in the KOOS symptoms subscale (r=0.5) (Table 4).

Baseline scores on perceived stress, mood, well-being, sleep quality, and overall QOL were significantly intercorrelated (r's ranging from 0.4 to 0.9). As illustrated in Table 5, improvements in mood, perceived stress, sleep quality, and both the mental and physical health components of QOL were likewise strongly interrelated at 2 months (r's from 0.4 to 0.8), with the strongest correlations observed between changes in mood and those in stress and the mental health composite score (r's from 0.7 to 0.8). Improvements in sleep quality were likewise correlated strongly with those in mood and perceived stress (r's=0.5). No statistically significant relationships were observed between changes in pain catastrophizing or kinesiophobia scores and any measure of psychosocial status.

## 4. Discussion

In this pilot RCT, participants assigned to both MM and ML demonstrated significant reductions in knee pain and overall OA severity. The MM group also showed significant improvements in multiple domains of knee function and greater gains than the ML group in two of these domains (knee-related QOL and sports/recreation-related function), as well as in overall (KOOS-WOMAC) score. Although criteria for minimal clinically important improvements (MCII) in the KOOS total score and in the KOOS pain and QOL subscales have not yet been established, improvements in the MM group in both the overall KOOS-WOMAC score and in all subscales far exceeded cutoffs proposed using a variety of different methods and anchors, including effect size (minimum 0.5) [70] and absolute change [71]. Similarly, the mean KOOS pain and function scores in the ML group also met or exceeded proposed cutoffs for MCIIs for knee pain and function, although effect sizes were substantially smaller overall than in the MM group. Using the more stringent criteria for the WOMAC pain and function subscales, the MM group achieved clinically significant improvement and an acceptable symptom state for pain and function, whereas the ML group did not [72, 73]. Likewise, only the MM group showed clinically important improvement in pain as measured by PGA, although both groups met criteria for clinically important reductions in the NRS [45, 74].

Although knee OA is a complex condition that remains incompletely understood [75], multiple interrelated factors likely contribute to the etiology and progression of OA. These include physical, neurobiological, and physiologic factors (e.g., altered neurologic structure and function, inflammation, joint degeneration, obesity, deterioration in muscular strength and cardiovascular fitness, and pain sensitization) as well as psychosocial and behavioral factors (e.g., sleep impairment, mood disturbance, low social support, pain-related fear, avoidant coping strategies, and sedentary behavior) [31, 76–78]. OA pain and dysfunction have been bidirectionally linked to distressful states and maladaptive behaviors, including psychologic stress [8, 79–81], depression and anxiety [79, 82–84], sleep impairment [7, 9, 85–87], fatigue [8, 84, 88], pain-related fear, [79, 89, 90], and catastrophizing [79, 91–94]. Consistent with both the biopsychosocial [95] and fear-avoidance models of knee OA [92, 96, 97], these reciprocal relationships contribute to a vicious cycle of increasing distress, sleep disturbance, fatigue, pain and pain sensitivity, sedentary behavior, and physical dysfunction, further amplifying risk for disability, morbidity, and mortality [8, 86, 91, 92, 98, 99]. Thus, therapies which address the key psychosocial dimensions of knee OA, in turn, strong determinants of OA pain and dysfunction, may be of particular benefit in the management of this serious and common chronic pain disorder.

To our knowledge, this is the first RCT to assess the effects of a simple meditation practice on pain, function, or related outcomes in patients with OA of the knee. Previous controlled studies in mixed and other pain populations suggest other meditation based interventions may improve pain and certain psychosocial outcomes in adults with musculoskeletal disorders, although observed effects have been modest overall. Notably, in a recent meta-analysis of 38 RCTs of mindfulness-based interventions in adults with chronic pain, including 3 studies with a small percentage (4-7%) of participants with OA or nonspecific arthritis, authors found low quality evidence for an overall small reduction in pain and for modest improvements in depression and QOL [28].

Likewise, this study is among the first to assess the effects of ML on OA pain and the first to evaluate potential benefits of ML for improving other outcomes of relevance to OA. Only one controlled trial has assessed the potential benefit of ML for OA, an RCT of 66 community-dwelling elders with symptomatic OA [100]. Consistent with our findings, elders randomized to a 14-day daily ML program showed significant reductions in pain over time relative to those randomized to daily quiet sitting [100]. Our findings are also in agreement with those from a recent meta-analysis of 14 RCTs of music listening (N=13) and other music interventions (N=1) for a range of chronic pain conditions, which indicated significant, moderate reductions in pain overall [27].

Observed improvements in OA pain and function in this study were also comparable or superior to those reported in studies of other nonpharmacologic therapies for knee OA, including acupuncture [101, 102], massage [103], yoga [40, 104–108],* t'ai chi* [109–111], and other forms of exercise [102, 112]. Notably, effect sizes for knee pain and function in the MM group were large (range 0.7-1.6), comparable to those reported for 8 weeks of physical therapy in patients with knee OA [41].

In addition, the reductions in pain and overall improvements in function observed in this study were also similar to or greater than those reported in previous trials of medications commonly prescribed for OA, including nonsteroidal anti-inflammatory drugs [113], acetaminophen [114], and opioids [113, 115]. Moreover, drug side effects can significantly mitigate the benefits of pharmaceuticals for OA management. For example, in a recent Cochrane review of oral and transdermal opioids for OA, the authors concluded that the small to moderate effects of these medications were outweighed by potential side effects and cautioned clinicians to discuss alternative treatments with patients [115]. Likewise, Machado et al. concluded that acetaminophen provides minimal short term benefit for OA, effects that are offset by significantly increased risk for elevated liver enzymes [114].

In this study, both the MM and ML groups also indicated significant improvements in perceived stress, overall mood, and QOL-Physical Health component. Relative to those assigned to ML, the MM group participants showed significantly greater improvement in mood and greater gains in QOL-MH. No published RCTs in OA patients have yet assessed the potential benefits of meditation or ML on psychosocial outcomes of relevance to OA. However, the improvements in mood and QOL observed in this study were similar to or greater than the effects documented in previous studies of yoga [116], tai chi [117, 118], physical therapy [117], exercise [112], and other nonpharmacologic therapies [119, 120] in adults with OA. Likewise, effect sizes in the MM group were comparable to or greater than those observed in controlled trials of mindfulness meditation [28] and music listening [27, 29] in other chronic pain populations.

In addition, those randomized to MM but not to ML showed significant improvements in sleep quality in this exploratory trial (p for between-group difference<0.04). Although there is evidence that music listening, meditation, yoga, and other mind-body and physical activity interventions may improve sleep in older adults [121–127], few trials of patients with OA or other chronic pain conditions have measured sleep as an outcome, and findings of these studies have been mixed [116, 128–131]. For example, evidence to date has indicated modest or no effects of yoga on sleep in OA [116] and inconsistent findings for the benefits of tai chi in another chronic pain syndrome, fibromyalgia [129–131].

In the current trial, both the MM and ML groups showed reductions in kinesiophobia, although the effect size was greater in the MM group. Pain catastrophizing was significantly reduced only in the ML group. Pain-associated catastrophizing and fear of movement are thought to contribute to the development and persistence of chronic pain; these factors may influence pain severity both directly and indirectly, and are significant predictors of physical performance and pain-related disability in those with chronic pain [79, 91, 92, 132, 133]. However, although kinesiophobia and pain-related catastrophizing have been recommended for inclusion in clinical trials of lifestyle interventions for OA [134], published RCTs regarding the effects of meditation, ML, yoga, or other mind-body interventions on these endpoints remain sparse. Moreover, findings from studies in other chronic pain populations have been mixed. In broad agreement with our findings, some studies suggest that certain yoga [135], mindfulness [136–139], and exercise-based interventions [140, 141] can be effective in lowering fear of movement [135, 136, 140, 141] and/or catastrophizing [137–139] in those with chronic pain, although other studies have reported modest or no effects [142–144].

*Possible Underlying Mechanisms*. While the mechanisms underlying the improvements in knee-related pain and function observed with MM and, albeit to a lesser extent, ML remain speculative, these simple mind-body therapies likely act via several pathways. Both meditation and music have been shown to decrease stress and stress reactivity, sleep impairment, anxiety, depression, fatigue, and sympathetic arousal [28, 121, 122, 145–151], factors linked to increased pain sensitivity and severity [8, 152–155]. As indicated above, there is a growing literature supporting the importance of sleep [9, 85, 86], mood [8, 84, 154], and other psychosocial factors [8, 79, 98] as both sequelae and determinants of OA associated pain, dysfunction, and disability. Consistent with these bidirectional relationships, improvements in knee pain, function, and other OA-related outcomes were positively correlated with improvements in mood, sleep, and QOL in this study.

Likewise, meditation and music may improve OA-related pain and function by reducing pain-associated fear of movement, a factor linked to the development and progression of chronic pain and associated disability, to the adverse mood and functional alterations associated with OA [132], and to the reluctance to engage in physical activity that is common among those with OA [90, 92, 156]. In this study, declines in fear of movement were greater in the MM than in the ML group and were strongly correlated with improvements in pain and function and, albeit more modestly, with those in mood, supporting a potential functional relationship. These findings suggest that reductions in kinesiophobia may in part mediate the observed beneficial effects of these simple mind-body practices on OA-related pain, function, symptoms, and mood changes, consistent with both the biopsychosocial and fear-avoidance models of chronic pain [92, 95–97, 132].

Finally, meditation and ML may also reduce pain by promoting beneficial functional and structural changes in brain structures associated with pain processing, attention, cognition, emotional regulation, and reward [157–165]. These alterations may, in turn, lead to a reduction in the central pain sensitization and hyperalgesia associated with OA [166–168]. For example, emerging evidence suggests that both meditation and ML can induce beneficial changes in central nervous system dopaminergic and other neurochemical systems [169–171] and enhance autonomic regulation, in part by modulating activation of the sympathoadrenal system and HPA axis and by increasing parasympathetic dominance [165, 172, 173]. In addition, recent controlled trials of ML and meditation suggest these practices can alter activity, increase grey matter density and/or volume, and promote functional connectivity in multiple brain areas involved in the cognitive, affective, and sensory processing of pain, including the periaqueductal grey matter, hippocampus, prefrontal cortex, insula, amygdala, orbitofrontal cortex, thalamus, somatosensory cortex, and anterior cingulate gyrus [157, 158, 163–165, 172, 174–180].

*Strengths and Limitations*. Strengths of the study include the community-based approach, rigorous, controlled design, and the use of multiple well-validated outcome measures of direct relevance to OA, including core outcomes recommended for knee OA trials of lifestyle/behavioral interventions [134]; treatment expectancies and program adherence were also measured. The two interventions were matched in terms of time, setting, and delivery. Both are easy-to-learn practices that can be performed readily in the home, with exit questionnaires suggesting high satisfaction with both programs. Baseline characteristics of the two groups were similar overall, indicating the randomization was successful in this study despite the small sample size. Retention and adherence were excellent and study satisfaction was high in both groups, further supporting feasibility of both the trial and the interventions.

This exploratory RCT also has several important limitations. The study did not include a long-term follow-up; thus, it was not possible to determine if the observed benefits were sustained over time. The sample size was small, reducing our ability to detect between-group differences and limiting generalizability. However, despite the limited power of the study, the MM group demonstrated both statistically and clinically significant improvements in all core outcomes and in four of the five secondary outcomes. Participants assigned to MM also demonstrated greater improvements than the ML group in certain domains of knee-related function/QOL, as well as in mood, sleep, QOL-MH, and fear of movement, outcomes of clear relevance to OA management. In addition, observed effect sizes in the MM group were comparable to or superior to those reported in RCTs of both nonpharmacologic [40, 41, 101–112] and pharmacologic interventions [113–115].

As this RCT did not include a usual care group, the relative influence of simple time trends or of the Hawthorne effect on change in outcomes could not be gauged. Likewise, due to the lack of an attention control, the influence of placebo effects cannot be ruled out. However, in this study, participant treatment expectancies (the primary determinant of placebo effects) were unrelated or negatively related to observed improvements; moreover, adjustment for treatment expectations* strengthened* between group differences in several measures, but otherwise did not appreciably affect our findings. Together, these findings suggest that placebo effects were unlikely to explain the improvements observed in this study. Although retention was high overall (91%), both drop-outs were in the MM group, potentially introducing bias. However, in both cases, withdrawal occurred early and for reasons unrelated to the intervention, and ITT analyses yielded results (both the within- and between-group) similar to those of our primary analyses, suggesting that the influence of differential attrition was likely to be minimal. Our study was also inadequately powered to assess the role of potential mediators, limiting conclusions regarding potential mechanisms. In addition, we did not include performance-based measures in this exploratory trial, and our study findings were thus reliant on self-reported measures of knee pain and function. However, given that patient perceptions and symptoms are primary drivers of the healthcare burden and disability associated with OA [181, 182], our findings are nonetheless clinically meaningful. The study population was predominantly female, non-Hispanic white, and older, limiting generalizability to other populations.

## 5. Conclusions

Findings of this exploratory RCT suggest that a simple MM and, possibly, ML program may be beneficial for reducing knee pain and dysfunction, decreasing stress, and improving mood, sleep, and QOL in older adults with knee OA, with improvements in knee function, mood, sleep, QOL-MH, and kinesiophobia that appeared greater in the meditation group. Improvements in mood, stress, sleep, QOL, and kinesiophobia were significantly correlated with improvements in several knee pain related outcomes, suggesting a potential mediating influence of these psychosocial factors. However, given the small size and exploratory nature of this RCT, our findings should be interpreted with caution. Larger controlled trials in multiethnic populations are clearly needed to confirm and extend these preliminary findings, to determine the cost-effectiveness of meditation versus other commonly used treatments for OA, and to investigate potential underlying mechanisms.

## Figures and Tables

**Table 1:** Participant baseline characteristics: pilot RCT of an 8-week mantra meditation (MM) and an 8-week music listening (ML) program in 22 adults with symptomatic osteoarthritis of the knee.

	**Overall (N=22)**		**MM (N=11)**		**ML (N=11)**		**P**
	**N**	%	**N**	%	**N**	%	
**Demographic characteristics**							
*Age (range 50-74 years)*							1.00
50-59 years	14	63.64%	7	63.64%	7	63.64%	
60+ years	8	36.36%	4	36.36%	4	36.36%	
*Mean±SE*	*58.46±1.37*		*58.09±1.60*		*58.82±2.23*		0.73
*Gender*							0.22
Female	15	68.18%	9	81.82%	6	54.55%	
Male	7	31.82%	2	18.18%	5	45.45%	
*Race/Ethnicity*							
Non-Hispanic White	18	81.82%	9	81.82%	9	81.82%	1.00
Minority	4	18.18%	2	18.18%	2	18.18%	
*Education*							0.37
12 years or less	2	9.09%	1	9.09%	1	9.09%	
Some post-high school education	8	36.36%	6	54.55%	2	18.18%	
4 years of college or more	12	54.55%	4	36.36%	8	72.73%	
*Mean±SE in years*	*15.46±0.42*		*15.09±0.64*		*15.82±0.57*		0.41
*Employment status*							0.72
Employed full time	15	68.18%	8	72.73%	7	63.64%	
Other	7	31.82%	3	27.27%	4	36.36%	
*Marital status*							0.54
Married/co-habiting	16	72.73%	7	63.64%	9	81.82%	
Divorced/Widowed	6	27.27%	4	36.36%	2	18.18%	
**Lifestyle and health-related factors**							
*Smoking status*							1.00
Never smoked	18	81.82%	9	81.82%	9	81.82%	
Ever smoker	4	18.18%	2	18.18%	2	18.18%	
*Caffeinated beverage consumption*							0.39
0-16 oz/d	14	63.64%	8	72.73%	6	54.55%	
17+ oz/day	8	36.36%	3	27.27%	5	45.45%	
*Mean oz consumed/day±SE*	*14.52±2.46*		*13.82±3.67*		*15.22±3.46*		0.78
*Physical activity*							0.04
None	9	40.91%	7	63.64%	2	18.18%	
10-149 min/week	6	27.27%	3	27.27%	3	27.27%	
150+ min/week	7	31.82%	1	9.09%	6	54.55%	
*Mean minutes/week±SE*	*148.64.64±50.74*		*129.09±96.57*		*168.18±37.53*		0.71
*Mean times/week±SE*	*3.07±0.63*		*2.00±0.88*		*4.14±0.81*		0.09
*Body mass index (BMI): Mean±SE*	*34.02±1.45*		*37.03±1.91*		*31.01±1.82*		0.03
*Obese*	15	68.18%	*9*	81.82%	6	54.55%	0.17
*History of diagnosed:*							
Diabetes	1	4.55%	0	0.00%	1	9.09%	0.72
Hypertension	15	68.18%	8	72.73%	7	63.64%	0.65
High cholesterol	7	31.82%	4	36.36%	3	27.27%	0.65
Depression	6	27.27%	4	36.36%	2	18.18%	0.34
Anxiety disorder	4	18.18%	2	18.18%	2	18.18%	1.00
Number of comorbid conditions							0.66
None	4	18.18%	2	18.18%	2	18.18%	
One	8	36.36%	3	27.27%	5	45.45%	
Two or more	10	45.45%	6	54.55%	4	36.36%	
Medications							
Analgesic Medications	15	68.18%	10	90.91%	5	45.45%	0.03
Opioids/Muscle relaxants	7	31.82%	3	27.27%	4	36.36%	0.45
NSAIDS	13	59.09%	9	81.82%	4	36.36%	0.03
Anti-depressant/anti-anxiety medications	8	36.36%	5	45.45%	3	27.27%	0.38
Anti-hypertensive medications	15	68.18%	8	72.73%	7	63.64%	0.65
Statins, other lipid-lowering medications	7	31.82%	3	27.27%	4	36.36%	0.65
Other medications^*∗*^	12	54.55%	6	54.55%	6	54.55%	0.72
Total medications							0.23
None	3	13.64%	0	0.00%	3	27.27%	
1-2	6	27.27%	3	27.27%	3	27.27%	
Three or more	13	59.09%	8	72.73%	5	45.45%	

^*∗*^Including medications for gastrointestinal reflux, diabetes, thyroid disorders, and osteoporosis.

**Table 2:** Mean baseline scores on osteoarthritis (OA) knee pain, function, and related outcomes and on sleep, stress, mood, well-being, quality of life, and other factors in a sample of older adults with symptomatic OA of the knee, stratified by treatment group.

***Outcomes***	**Mantra Meditation (N=11)**	**Music Listening (N=11)**	**P**
	***Mean (SE)***	***Mean (SE)***	
**Knee Pain-Related (Core) Outcomes**			
*Knee Injury and OA Outcome Score (KOOS)*			
Total-WOMAC*∗*(range 0-240)	106.64 (6.54)	119.73 (12.70)	0.37
Pain (range 0-90)	41.91 (2.41)	49.82 (4.38)	0.16
Pain-WOMAC (range 0-50)	20.90 (0.96)	25.82 (2.92)	0.14
Symptoms (range 0-70)	32.64 (2.90)	33.73 (3.09)	0.89
Stiffness-WOMAC (range 0-20)	10.82 (0.93)	12.45 (1.03)	0.25
Function (range 0-170)	74.90 (5.57)	81.46 (9.43)	0.56
Sports/Recreation (range 0-50)	35.73 (3.58)	34.91 (4.08)	0.88
Quality of Life (range 0-40)	29.82 (1.83)	29.18 (2.45)	0.84
*Numeric Rating Scale (range 0-10)*			
Pain now	3.90 (0.50)	4.27 (0.69)	0.67
Average pain (last week)	4.91 (0.31)	5.91 (0.56)	0.15
Worst pain (last week)	7.20 (0.57)	7.82 (0.0.62)	0.47
Least pain (last week)	2.80 (0.36)	2.73 (0.0.78)	0.93
*Patient Global Assessment (range 0-10)*	6.00 (0.36)	6.36 (0.53)	0.57
**Secondary Outcomes**			
***Stress, Sleep Quality, Mood, and Well-being***			
*Perceived Stress Scale*	17.30 (3.24)	15.33 (1.32)	0.25
*Pittsburgh Sleep Quality Index*	9.78 (3.24)	8.09 (2.21)	0.23
*Profile of Mood States (total score)*	30.90 (11.19)	29.37 (11.19)	0.93
*Psychological Well-being Scale*	84.73 (3.70)	82.64 (5.05)	0.74
***Health related Quality of Life (SF-36)***			
*Mental Health Composite Score*	60.20 (6.92)	70.00 (6.43)	0.31
*Physical Health Composite Score*	45.74 (4.85)	47.90 (5.62)	0.77
**Potential mediators**			
Tampa Scale for Kinesiophobia	17.30 (1.02)	16.82 (0.76)	0.71
Pain Catastrophizing Scale	15.10 (3.92)	22.27 (3.98)	0.21
**Treatment Expectancy(CEQ)**			
How confident that tx will be beneficial (1-10)	7.73 (0.41)	7.82 (1.25)	0.78
Degree improvement expected (1-10)	7.00 (0.49)	7.27 (0.45)	0.96

^*∗*^Calculated as the sum of the WOMAC Pain, Stiffness, and Function subscale scores.

CEQ= Credibility Expectancy Questionnaire (higher numbers indicate higher expectancy).

**Table 3:** Change over time in knee pain, function, perceived stress, sleep, mood, and related outcomes in older adults with knee osteoarthritis assigned to a mantra meditation or music listening program.

	**Mantra Meditation**			**Music Listening**			
	**Change at 2 months**			**Change at 2 months**			
	**(Mean ± SE)**	**P** ^**∗**^	**ES**	**(Mean ± SE)**	**P** ^**∗**^	**ES**	**P** ^ŧ^
**Knee Pain-Related (Core) Outcomes**							
* KOOS*							
* *Total (WOMAC)^*∗∗*^	-38.00 (12.27)	0.015	2.2	-19.00 (8.82)	0.06	0.5	0.07
* *Pain	-13.33 (4.45)	0.02	0.7	-9.36 (3.77)	0.03	0.7	0.16
WOMAC pain	-8.00 (2.74)	0.02	2.3	-4.82 (2.47)	0.08	0.5	0.26
* *Symptoms	-5.22 (2.67)	0.08	0.7	-2.55 (1.98)	0.23	0.3	0.42
* * Stiffness (WOMAC)	-2.00 (1.35)	0.18	0.4	-1.91 (0.78)	0.03	0.5	0.95
* *Function (ADL)	-28.00 (8.91)	0.02	0.8	-12.27 (6.67)	0.10	0.4	0.45
Sports/Recreation	-17.00 (7.06)	0.04	0.9	-2.91 (4.96)	0.57	0.2	0.09
* *Knee-related Quality of Life	-8.67 (3.19)	0.03	0.8	-1.91 (1.52)	0.24	0.2	0.06
* Numeric Rating Scale*							
* *Knee pain now	-1.63 (0.50)	0.01	1.0	-1.73 (0.66)	0.03	0.8	0.58
* *Average knee pain	-2.13 (0.79)	0.03	1.6	-1.45 (0.47)	0.01	0.8	0.90
* *Worst knee pain	-1.38 (0.53)	0.04	0.7	-1.18 (0.50)	0.12	0.6	0.94
* *Least knee pain	-1.63 (0.42)	0.01	1.3	-1.64 (0.66)	0.03	0.6	0.65
* Patient Global Assessment*	-2.00 (0.87)	0.025	1.0	-1.18 (0.50)	0.04	0.7	0.19
**Secondary Outcomes**							
***Stress, mood, well-being and sleep quality***							
* Perceived Stress Scale*	-4.78 (2.00)	0.04	0.7	-3.45 (1.08)	0.01	0.4	0.56
* Profile of Mood States (total score)*	-34.11 (9.64)	0.008	1.5	-10.91 (5.19)	0.06	0.2	0.04
* Psychological Well-being Scale*	4.00 (3.09)	0.23	0.4	2.45 (2.44)	0.34	0.2	0.74
* Pittsburgh Sleep Quality Index (total score)*	-2.38 (0.80)	0.02	0.7	-0.36 (0.49)	0.48	0.1	0.04
***Health related Quality of Life (SF-36)***							
* Mental Health Component*	17.96 (5.61)	0.01	0.9	5.30 (3.63)	0.18	0.2	0.07
* Physical Health Component*	20.69 (5.11)	0.004	1.0	11.59 (3.76)	0.01	0.5	0.16
**Potential Mediators**							
* Tampa Scale for Kinesiophobia-6 (total)*	-4.22 (1.53)	0.03	1.1	-1.45 (0.65)	0.05	0.7	0.09
* Pain Catastrophizing Scale Total*	-4.78 (2.48)	0.09	0.5	-7.64 (2.24)	0.007	0.7	0.25

^*∗*^Repeated measures ANOVA (RM) ŧ Between group difference at 2 months, adjusted for age.

^*∗∗*^Calculated as the sum of the WOMAC Pain, Stiffness, and Function subscale scores.

**Abbreviations:** ES= effect size; KOOS=Knee Injury and Osteoarthritis Outcome Score; SE=standard error.

**Table 4:** Relation of changes over time in knee pain-related outcomes to mood, sleep, well-being, and quality of life and to pain-related fear and catastrophizing in older adults with symptomatic osteoarthritis of the knee.

**Change from baseline**	**Change over time at 2 months**										
	**KOOS Total-WOMAC**	**KOOS Function**	**KOOS Symptoms**	**KOOS Pain**	**KOOS Sport**	**KOOS QOL**	**NRS Pain now**	**NRS Pain average**	**NRS Pain worst**	**NRS Pain least**	**PGA**
**Stress, sleep, mood, and QOL**											
*Perceived stress (PSS)*					**0.39(** **∗** **)**						
*Mood (Profile of Mood States: total score)*	**0.38(** **∗** **)**	**0.42(** **∗** **)**					**0.4**7^**∗**^				**0.39(** **∗** **)**
*Sleep Quality (Pittsburgh Sleep Quality Index)*											
*Total Score*							**0.41 (** **∗** **)**			**0.40(** **∗** **)**	
Mean number hours sleep			**-0.40(** **∗** **)**				**-0.4**6^**∗**^	**-0.4**7^**∗**^		**-0.5**2^**∗∗**^	
*Health related Quality of Life (SF-36)*											
Mental Health component	**-0.5**3^**∗∗∗**^	**-0.6**1^ŧ^				**-0.4**6^**∗**^	**-0.4**3^**∗**^				**-0.4**9^**∗∗**^
Physical Health component	**-0.5**3^**∗**^	**-0.5**3^**∗∗**^				**-0.39(** **∗** **)**	**-0.4**4^**∗**^	**-0.4**7^**∗**^			**0.39(** **∗** **)**
**Pain-related Fear/Catastrophizing**											
*Tampa Scale Kinesiophobia (total score)*	**0.5**2^**∗∗**^	**0.5**2^**∗∗**^	**0.5**1^**∗∗**^	**0.41(** **∗** **)**	**0.4**4^**∗**^	**0.63†**		**0.5**6^**∗∗∗**^			**0.5**5^**∗∗∗**^
*Pain Catastrophizing Scale (total score)*			**0.5**4^**∗∗∗**^								

Abbreviations: NRS=Numerical Rating Scale; PGA=Patient Global Assessment; QOL=quality of life.

(*∗*) p<0.1, ^*∗*^p<0.05; ^*∗∗*^p <0.025; ^*∗∗∗*^p<0.01; ^ŧ^p<0.005.

**Table 5:** Relation between changes over time in mood, sleep, perceived stress, and quality of life in adults with knee osteoarthritis.

Change from baseline	Change over time at 2 months				
	Mood	Perceived Stress	Sleep Quality	QOL, Mental Health	QOL, Physical Health
Mood (Profile of Mood States)		0.66^ŧ^	0.54^*∗∗*^	-0.75^ŧŧ^	-0.57^*∗∗∗*^
Perceived stress (Perceived Stress Scale)	0.66^ŧ^		0.48^*∗*^	-0.44^*∗*^	-0.56^*∗∗∗*^
Sleep quality (Pittsburgh Sleep Quality Index)	0.54^*∗∗*^	0.48^*∗*^			
Health-related OOL (SF-36)					
Mental Health Component	-0.75^ŧŧ^	-0.44^*∗*^			
Physical Health Component	-0.57^*∗∗∗*^	-0.56^*∗∗∗*^			

^*∗*^p <0.05; ^*∗∗*^P<0.025; ^*∗∗∗*^p<0.01; ^ŧ^p<0.005; ^ŧŧ^p<0.001.

**Abbreviations: **QOL=quality of life.

## Data Availability

The data used to support the findings of this study are available from the corresponding author upon request.
